# Late renal recovery after treatment over 1 year post-onset in an atypical hemolytic uremic syndrome: a case report

**DOI:** 10.1186/s12882-020-01897-4

**Published:** 2020-06-22

**Authors:** Yusuke Kuroki, Koji Mitsuiki, Kaneyasu Nakagawa, Kazuhiko Tsuruya, Ritsuko Katafuchi, Hideki Hirakata, Toshiaki Nakano

**Affiliations:** 1 Nephrology & Dialysis Center, Japanese Red Cross Fukuoka Hospital, 3-1-1, Ogusu, Minami-ku, Fukuoka, 815-8555 Japan; 2 Department of Nephrology, Nara Medical University, Nara, Japan; 3 Division of Nephrology, National Hospital Organization Fukuokahigashi Medical Center, Koga, Japan; 4 Department of Medicine and Clinical Science, Graduate School of Medical Sciences, Kyushu University, Fukuoka, Japan

**Keywords:** Atypical hemolytic uremic syndrome, Mesangiolysis, Late renal recovery, Platelet normalization, Eculizumab

## Abstract

**Background:**

Atypical hemolytic uremic syndrome (aHUS) is a life-threatening disease that leads to end-stage kidney disease if only a poor response to plasma exchanges (PEs) or eculizumab therapy is achieved.

**Case presentation:**

A 58-year-old Japanese man presented with thrombocytopenia, anemia, and kidney failure requiring dialysis without any underlying disease. A kidney biopsy revealed marked mesangiolysis in all glomeruli, compatible with thrombotic microangiopathy (TMA). Based on the positive anti- factor H antibody and negative result for secondary TMA, we diagnosed him as aHUS. Despite eculizumab administration after eight sessions of PE, neither platelet normalization nor kidney recovery was achieved. Eight months later, we discontinued eculizumab therapy due to anaphylactic reaction. At 15 months after the onset of TMA, his platelet count increased gradually from 40 to 150 × 10^3^/μL with a decreased serum creatinine level and increased urine output, eventually allowing the withdrawal of dialysis therapy. A second kidney biopsy showed mesangial widening compatible with the healing of TMA.

**Conclusions:**

This case indicates that aHUS with PEs and eculizumab therapy has the potential for renal recovery even if over 1 year has passed.

## Background

The pathophysiology of thrombotic microangiopathy (TMA) has been described as consumptive thrombocytopenia, microangiopathic hemolytic anemia, and a manifestation of end-organ ischemia such as kidney failure [1]. Thrombotic thrombocytopenic purpura (TTP) is defined as decreased von Willebrand factor cleaving protease (a disintegrin-like and metalloproteinase with thrombospondin type 1 motifs 13; ADAMTS13) activity [2]. However, it is sometimes difficult to distinguish TMA from other diseases by only the clinical presentation and laboratory data. A kidney biopsy is indicated to confirm the diagnosis of TMA and evaluate the patient’s prognosis, although it is difficult to perform a kidney biopsy in patients with thrombocytopenia [3]. The pathological findings of TMA are characterized by fibrin or platelet thrombi in the glomerular capillary lumina and arterioles, thickening of the capillary wall and arterioles, mesangiolysis, and extensive widening of the subendothelial space due to endothelial injury [4, 5].

Most cases of hemolytic uremic syndrome (HUS) are caused by a Shiga toxin-producing *Escherichia coli* (STEC) infection [6]. HUS without a STEC infection is classified as atypical HUS (aHUS), resulting from a dysregulation of an alternative complement pathway due to genetic abnormalities or other unknown reasons [7]. Activation of the complement system eliminates the thromboresistance function of endothelial cells, leading to systemic thrombosis. Over 50% of patients with aHUS will never recover kidney function; the response rate to the treatment differs with each genetic abnormality [8].

Eculizumab, a humanized monoclonal antibody that blocks the cleavage of complement 5, is effective in aHUS [9], however some cases for which eculizumab was not effective have been reported [10]. The optimal schedule for the discontinuation of eculizumab therapy in responsive cases was recently discussed [11], but a schedule for poor-response cases has not been established.

Here we describe the case of a patient who suddenly developed aHUS with kidney failure requiring hemodialysis, after 15 months of hemodialysis therapy renal recovery with platelet normalization was confirmed, and the patient’s hemodialysis was then discontinued.

## Case presentation

A 58-year-old Japanese man was admitted to our hospital with the complaints of fever, pedal edema and facial puffiness that had begun 6 days before his admission. Six months earlier, his annual medical checkup showed normal blood pressure, no proteinuria, and an estimated glomerular filtration rate of 68.2 mL/min/1.73 m^2^. He had no history of loose stool, diarrhea or consumption of uncooked meat. He had no similar history including his family such as impaired kidney function. He was not currently taking any medications, health foods or supplements. His work and hobbies did not expose him to radiation or harmful substances, and he did not use recreational drugs.

On admission, he was conscious and had facial puffiness and bilateral pitting pedal edema. His blood pressure was 150/80 mmHg. His urine examination showed proteinuria and hematuria with a urinary protein to creatinine ratio (UP/UCr) of 1.2 g/g Cr. This UP/UCr remained at 0.5–1.2 g/g Cr after admission. His hemoglobin and platelet count declined within 2 weeks from 10.3 to 6.9 g/dL and from 106 to 48 × 10^3^/μL, respectively. His albumin level was 3.0 g/dL, and decreased to 2.2 g/dL. The LDH level was 296 IU/L, and increased to 341 on the 4th day. The LDH level remained in the range of 263–341 IU/L. His haptoglobin level was 36 mg/dL, which was the lower limit of the normal range. Peripheral blood smears indicated that 3–5% of red blood cells were schistocytes. Serum levels of complement 3 and 4 were within normal ranges. Other immunological tests including anti-nuclear antibody, anti-dsDNA antibody, myeloperoxidase anti-neutrophil cytoplasmic antibody, proteinase 3 anti-neutrophil cytoplasmic antibody, anti-glomerular basement membrane antibody and anti-lipopolysaccharide antibody showed no abnormalities. Both the direct and indirect erythrocytes antiglobulin tests were negative. Examination of plasma protein fractionation showed no monoclonal gammopathy.

**Fig. 1:**
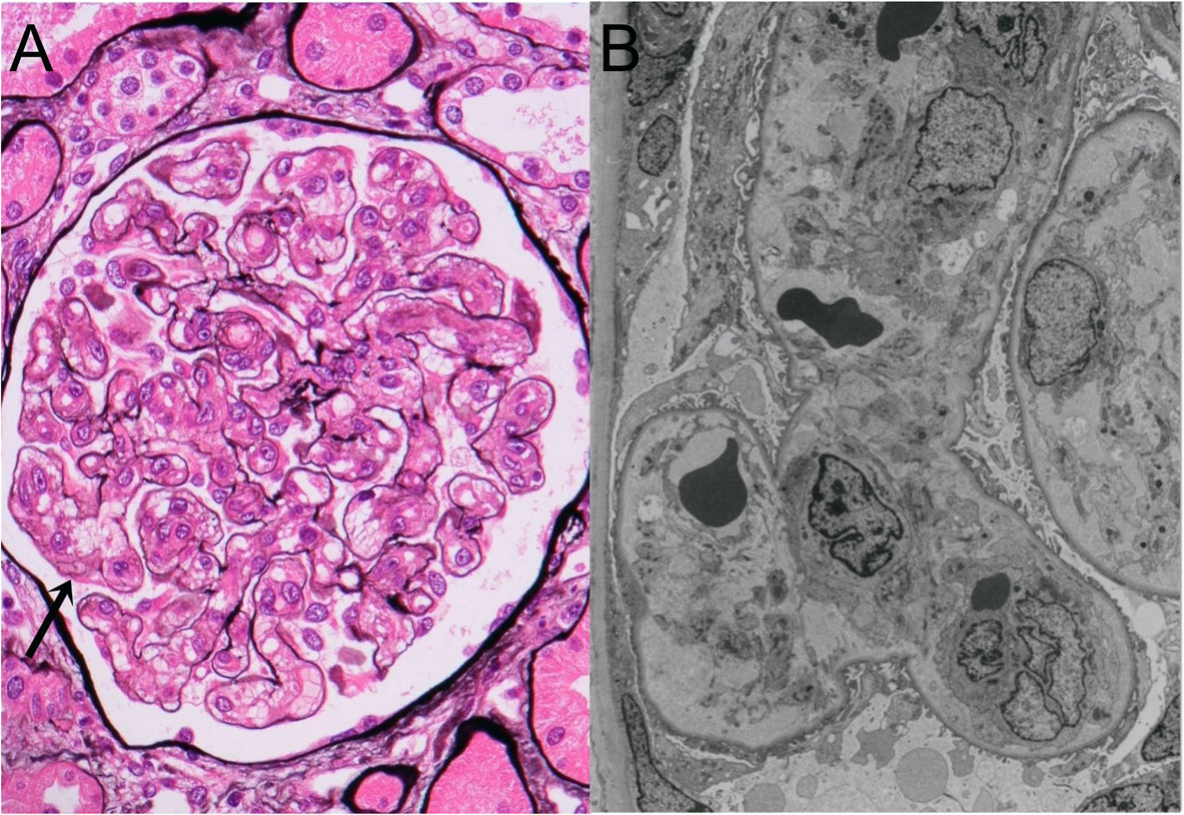
**a** Light microscopy: Marked mesangiolysis was seen in all glomeruli. An unanchored dilated capillary had developed as result of the mesangiolysis (arrow). No fibrin or platelet thrombi were observed in the capillary lumina (Periodic acid-methenamine-silver stain, × 400). **b** Electron microscopy: Widening of the subendothelial space and swelling of the endothelial cells resulted in thickening of the capillary wall, leading to narrowing of the capillary loop. Extravasations of red blood cells to the subendothelial space were seen. (× 5750)

The patient’s serum creatinine level rapidly increased within 3 weeks from 1.6 to 4.8 mg/dL. A kidney biopsy showed marked mesangiolysis and subendothelial swelling in 16 of 18 glomeruli contained in the sections, with the remaining 2 glomeruli being obliterated by global sclerosis (Fig. 1). Any thrombi, fibrinoid necrosis and hemoglobin casts were not identified. By immunofluorescence, immunoglobulin, complement and fibrinogen were all negative. These findings were compatible with the definition of TMA. The patient’s ADAMTS13 activity decreased to 30% with negative ADAMTS13 inhibitor but not to a level as low as that observed in TTP.

The result of the laboratory work-up for secondary TMA was negative such as anti-phospholipid antibody syndrome, scleroderma, malignant hypertension, human immunodeficiency virus infection and homocysteinemia. In regard to etiologic analyses, no gene mutation such as *CFH, MCP, CFI, THBD, C3* and *CFB* was identified. Anti-factor H antibody was detected by a Western blotting analysis and an enzyme-linked immunosorbent assay (ELISA) with a titer of 76.9 AU/mL. This level was not as high as the levels in other anti-factor H antibody-positive cases. However, because both Western blotting and ELISA were positive, we concluded that the patient was anti-factor H antibody-positive. Although hemolytic assay of sheep erythrocytes was negative of 9.6% and complement factor H-related (CFHR) proteins 1 and 3 were not deleted, it seemed likely that this case was anti-factorH antibody-associated aHUS.

**Fig. 2:**
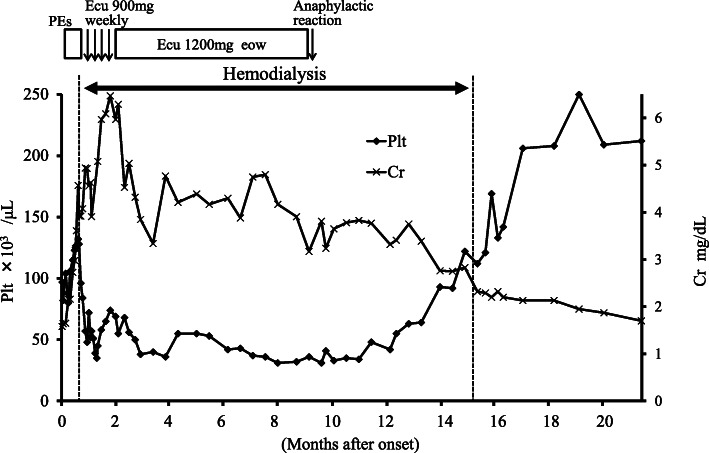
The clinical course of the patient, a 58-year-old Japanese man. Changes in serum creatinine and platelet count over time. During the regular dialysis therapy, serum creatinine was measured prior to dialysis sessions at 2-day intervals. PEs, plasma exchanges; Ecu, eculizumab; eow, every other week; Plt, platelet count; Cr, serum creatinine

Plasma exchanges (PEs) and hemodialysis therapy were started on the 11th day after the patient’s admission. After eight PEs, we initiated treatment with eculizumab 900 mg/week for 4 weeks and after that every other week (Fig. 2). As his kidney function deteriorated, his urine volume gradually decreased until he became oliguric when starting hemodialysis. Soon after starting hemodialysis, he became anuric. The hematologic abnormality and kidney failure requiring dialysis persisted. The platelet count remained 40 × 10^3^/μL. After 8 months of eculizumab administration, the patient suddenly developed a generalized rash with itching and slight dyspnea during the eculizumab administration. Because an anaphylactic reaction was suspected, we therefore discontinued the maintenance eculizumab therapy with his consent.

We performed an analysis of *C5* gene for his poor response to eculizumab, because its single missense heterozygous mutation causes a poor response to eculizumab in Japanese patients with paroxysmal nocturnal hemoglobinuria (PNH) [12]. However, the analysis did not detect this mutation.

At 15 months after the onset of aHUS, the patient’s platelet count had increased gradually from 40 to 150 × 10^3^/μL. At the same time, the serum creatinine level before the hemodialysis session had declined with increased urine output despite the persistence of anuria after starting dialysis (Fig. 2). We were able to discontinue dialysis therapy, and his creatinine level eventually declined to 1.7 mg/dL.

**Fig. 3:**
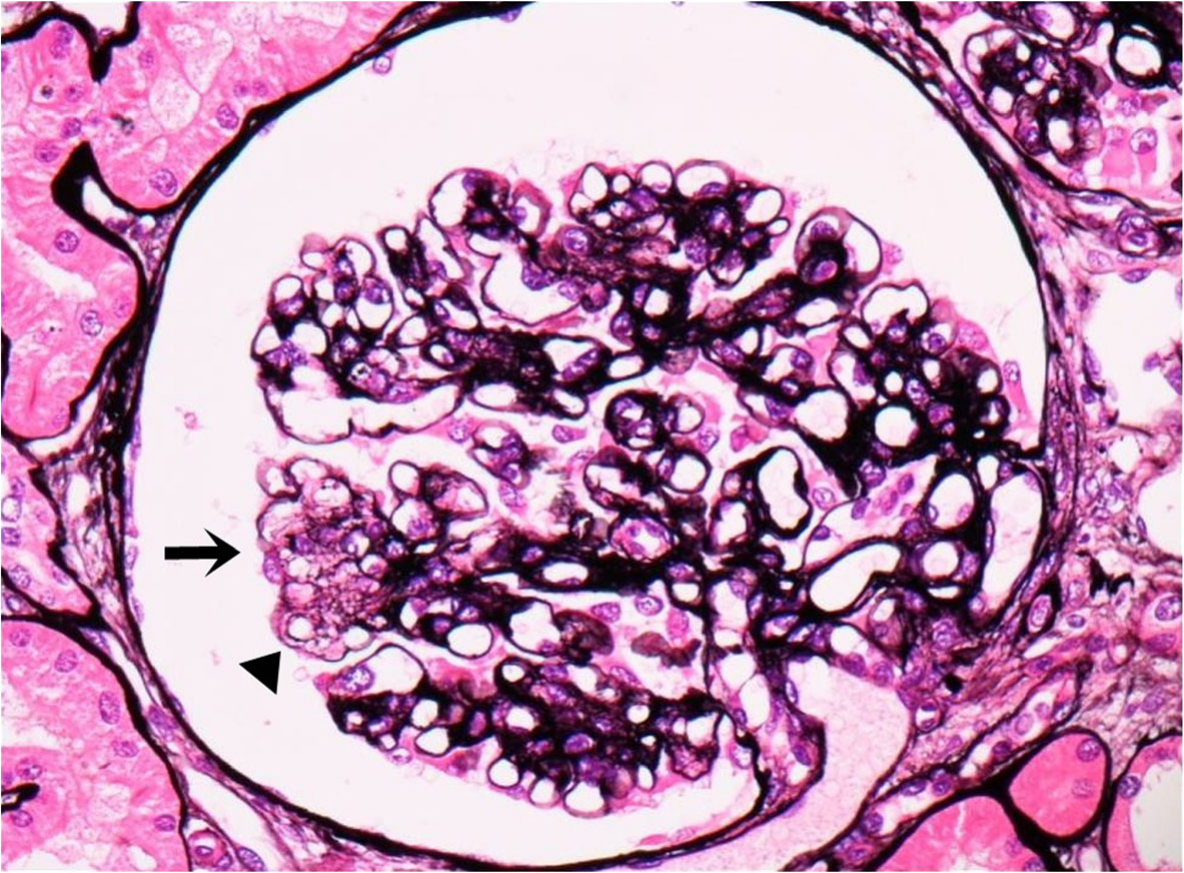
Light microscopy: The glomeruli showed mesangial widening and thick capillary walls. Mesangiolysis (arrow) and subendotherial swelling with double contour (arrowhead) were segmentally seen in some glomeruli. (Periodic acid-methenamine-silver stain, × 400)

We performed a second kidney biopsy and found that mesangiolysis markedly decreased. Mesangiolysis and subendotherial swelling with double contour of the glomerular basement membrane were only segmentally observed in some glomeruli. Mesangial widening due to increase in matrix was also observed in most glomeruli (Fig. 3). These findings were compatible with the healing stage of TMA.

Thus, our patient developed aHUS requiring dialysis with positive anti-factor H antibody and negative CFHR protein 1 and 3, and the aHUS did not improve rapidly despite treatment with PEs and eculizumab. However, 15 months later he had a recovery of kidney function with platelet normalization and his dialysis therapy was discontinued.

## Discussion and conclusion

It is suspected that approximately 60% of aHUS cases are caused by a gene mutation in complement system [7] and that 5% of the cases are due to anti-factor H antibody [13]. The development of anti- factor H antibody is strongly associated with deletion of both CFHR proteins 1 and 3, which are CFHR proteins that bind to the central complement component C3b [14], coding for proteins showing sequence and structural homology to factor H in close proximity to *CFH* gene [15]. This deletion results from genomic rearrangement in *CFH-CFHR* region. In our patient, anti-factor H antibody was detected by a Western blotting analysis and ELISA despite no deletion of CFHR proteins 1 and 3 protein. Moore et al. reported that patients with CFHR protein 1 deletion had 0 copy number of *CFHR1* and patients with CFHR protein 1 had a normal copy number of *CFHR1* [16]. Considering the above findings, it seemed likely that this case was anti-factor H antibody associated aHUS without abnormality of *CFH-CFHR1–5* genomic region.

In our patient, eculizumab improved neither the platelet normalization nor the kidney recovery despite its administration 5 weeks after the onset. Furthermore, our patient showed negative hemolytic assay of sheep erythrocyte. These findings suggest that the development of aHUS may be resulted from another mechanism in addition to anti factor H antibody. Fakhouri et al. reported that causes of poor response to eculizumab were severe unrecognized pathologic features of renal TMA, gene abnormalities carrying a high risk of end-stage renal disease, a delay in the initiation of eculizumab, and a relatively short duration of eculizumab treatment [10]. It is possible that an unrecognized genetic mutation or pathophysiology was the cause of our patient’s poor response to treatment. Further, earlier administration might have led to a better clinical course, though it took time to exclude TMA other than aHUS.

We did not administer immunosuppressive therapy for anti-factor H antibody. Several months had already passed by the time we concluded that the patient was positive for anti-factor H antibody. The anti-factor H antibody-related HUS showed a good response to treatment in the short term, but a poor renal outcome over the long term [17]. We considered that it was hopeless to recover kidney function due to the amount of time passed since the development of ESKD. Immunosuppressive medications were considered too risky in terms of immunocompromise, and so they were not administered.

In terms of PNH, Japanese patients with a single missense C5 heterozygous mutation have shown a poor response to eculizumab [12]. The prevalence of this polymorphism with PNH (3.2%) is similar to that among healthy Japanese people (3.5%) [12]. We therefore speculated that our patient might have a polymorphism of C5 gene, but it was not identified. Although this polymorphism was not detected in the present case, unidentified gene abnormalities may be related to no rapid response to eculizumab.

Kidney recovery with platelet normalization occurred more than 1 year after the onset of aHUS in our patient. To our knowledge, among the reports of treatment-resistant aHUS patients requiring dialysis, there has been no reports of cases with kidney recovery years later, allowing dialysis discontinuation. In a trial examining an adult aHUS population treated with eculizumab, 73% showed a complete TMA response with eculizumab and 79% discontinued dialysis during eculizumab treatment [18]. However, the poor-response cases in that trial needed dialysis therapy. In this case, it is possible that the 8 months of eculizumab therapy protected his kidneys and influenced his subsequent recovery, although he showed a poor response just after eculizumab administration. In light of this case, the timing of eculizumab discontinuation should be considered not only in rapid responsive cases, but also in poor-response cases.

In conclusion, this case indicates that aHUS has the potential for renal recovery with platelet normalization even over 1 year post onset. This recovery may be associated with PEs and eculizumab administration. Clinicians should thus keep in mind the possibility of renal recovery many months later.

## Data Availability

The datasets used and/or analyzed during the current study available from the corresponding author on reasonable request.

## Abbreviations

TMA: Thrombotic microangiopathy
TTP: Thrombotic thrombocytopenic purpura
ADAMTS13: A disintegrin-like and metalloproteinase with thrombospondin type 1 motifs 13
HUS: Hemolytic uremic syndrome
STEC: Shiga toxin-producing *Escherichia coli*
aHUS: atypical hemolytic uremic syndrome
ELISA: Enzyme-linked immunosorbent assay
CFHR: Complement factor H-related
PEs: Plasma exchanges
PNH: Paroxysmal nocturnal hemoglobinuria (PNH)
